# Knowledge of cytomegalovirus infection among women in Saudi Arabia: A cross-sectional study

**DOI:** 10.1371/journal.pone.0274863

**Published:** 2022-09-29

**Authors:** Ali A. Almishaal

**Affiliations:** College of Applied Medical Sciences, University of Hail, Hail, Saudi Arabia; University of Arkansas for Medical Sciences, UNITED STATES

## Abstract

**Background:**

Cytomegalovirus (CMV) is the most common intrauterine congenital infection in humans. Worldwide seropositive rates of CMV are considerably high among women of childbearing age. There is currently no optimal drug treatment nor a vaccine for congenital CMV infection and therefore the best available program to date of prevention is practicing standard hygienic measures. The success of this program relies on women’s knowledge of CMV modes of transmissions, and risk and preventative behaviors.

**Objective:**

The current study aims to assess the awareness and knowledge of CMV infection among women in Saudi Arabia.

**Method:**

In this cross-sectional study, an online self-administered questionnaire was distributed to women 18 years of age or older residing in all regions of Saudi Arabia. The questionnaire included questions to assess awareness of CMV, knowledge of symptoms, transmission, and preventative measures of CMV infection.

**Results:**

Out of the 1004 women who completed the questionnaire, self-reported knowledge of CMV was considerably low with only 82 women (8.17%) having heard of CMV infection. Most women reported learning about CMV from the internet and university. In binary logistic analyses, women pursuing studies in healthcare and those working in health professions, as well as those with undergraduate and graduate college degrees were significantly associated with higher knowledge of CMV. Urban area of residence was significantly associated with lower levels of knowledge of CMV. Among women who reported they had heard of CMV, their knowledge of CMV modes of transmission, symptoms, and preventative measures was considerably low. Regarding the transmission route, 23% reported no knowledge of modes of transmission, 59.75% reported mother-to-child transmission, 48.78% reported sexual intercourse, and 45% reported contact with body fluids of an infected person. Regarding knowledge of symptoms of congenital CMV, mental retardation and death were the most commonly reported clinical presentations.

**Conclusion:**

The current study showed that the overall knowledge of CMV is very low among women in Saudi Arabia. Working in the healthcare field and higher education levels were significantly associated with better knowledge of CMV. It is crucial that women are provided with information regarding CMV-associated complications and preventative measures against mother-to-fetus transmission of CMV.

## Introduction

Human Cytomegalovirus (CMV), a member of the beta-herpesvirus family, is a very common virus that can infect almost anyone of all age groups. CMV is a major global health problem and is recognized as the most common intrauterine viral infection in the world [1]. CMV virus remains latent in many body cells for lifetime following primary infection. In otherwise healthy individuals, CMV does not cause serious medical problems. However, serious infections are usually limited to infants who become infected before birth and in people with a weakened immune system, for example, people with acquired immunodeficiency syndrome or who have undergone an organ transplant.

CMV is transmitted from person-to-person via close direct contact with bodily fluids of the infected person including saliva, urine, breast milk, and blood. CMV can also be transmitted through the placenta from an infected mother to her unborn baby or the baby can become infected during childbirth (congenital CMV). Such maternal-to-child transmission occurs either in the case where a pregnant woman contracts a primary infection during pregnancy or in the case of CMV reactivation or reinfection with a different CMV strain during pregnancy. Seronegative pregnant women, for example, are at higher risk of contracting CMV through close contact with young children [2–4]. This high-risk stems from the evidence that CMV can persist in children’s urine and saliva for months after contracting the infection peaking at 2 years of age, while these children are rarely symptomatic [5–7]. Previous studies have shown that CMV is frequently transmitted from young children in daycare centers to their seronegative parents [5], while many mothers working in day care centers seroconvert during the first year of work [5]. This represents a highly likely major source for increased rate of CMV maternal-to-fetus transmission among women of reproductive age.

CMV is considered as the most common viral infection that infants are born with accounting for approximately 0.64–4% among live-born infants [8, 9]. Previous studies suggest that more children are affected by congenital CMV than other well-recognized medical problems such as spina bifida and Down syndrome [10]. Of the CMV-infected newborns, 85–90% are asymptomatic while the remaining 10% are symptomatic at birth or may progressively develop permanent disabilities [11]. The long-term impacts of congenital CMV include sensorineural hearing loss, visual problems, intellectual disability, cerebral palsy, and other forms of neurodevelopmental disabilities [12]. Indeed, congenital CMV infection represents the most common viral cause of permanent sensorineural hearing loss in children [10, 13, 14].

Seroprevalence estimates from previous studies worldwide indicated a high rate of seropositivity of CMV antibodies among women, ranging from 50 to 100% [15]. Similarly, high rates of CMV seropositivity were reported among women in Saudi Arabia [16–20]. In the context of Saudi Arabia, it is a common practice to screen for human CMV among women with a history of pregnancy complications, those exhibiting intrauterine growth restriction during pregnancy, or recurrent abortions as part of a screening protocol for TORCH congenital infections [synonym for Toxoplasmosis, Other agents, Rubella, CMV, Herpes Simplex Virus]. Many serological surveys in Saudi Arabia have reported seroprevalence rates of these TORCH infections with the prevalence of CMV being the highest compared to other TROCH infections [27.4–35% for *Toxoplasma gondii*, 90.9–94.7-% for herpes simplex type 1 (HSV-1), 0.5–27.1% for herpes simplex type 2 (HSV-2), 92.1–100% for CMV, and 88.9–93.3% for rubella] [16, 18]. In contrast to the high prevalence rates of TORCH infections, knowledge of these infections is considerably low among Saudi women. For example, more than half of Saudi women had very low level of knowledge about Toxoplasmosis [21–23] and rubella infection [24] adverse effects during pregnancy. Moreover, there are currently no CMV awareness campaigns in Saudi Arabia to provide pregnant women in particular with relevant information about how to prevent CMV transmission to their unborn baby. This lack of awareness of CMV infection and its preventative measures may lead to higher rates of pregnant women inadvertently transmitting the virus to their unborn babies. There is currently no cure for CMV infection as there is insufficient scientific evidence to recommend a treatment, nor is there an effective vaccine to prevent or treat CMV infection during pregnancy. This suggests that practicing the recommended hygiene measures is currently the best method to prevent CMV infection during pregnancy. This can be achieved through providing adequate knowledge about CMV infection, its risk factors, and preventative measures to pregnant women or those planning pregnancy. Many studies have been performed to assess the level of awareness and knowledge of CMV worldwide. A common finding of most studies showed that most women are unaware (61–87%) of CMV and its primary preventative measures that need to be practiced to reduce the likelihood of contracting CMV [11, 25–30]. This is in contrast to other well-known diseases that can potentially cause serious medical problems to the fetus if contracted during pregnancy such as TORCH infections.

Despite the high prevalence rates of CMV in Saudi Arabia, there is a scarcity of data on the awareness and knowledge regarding CMV infection and its symptoms, modes of transmission, and prevention among women in the country. Therefore, we conducted a cross-sectional survey study to determine the awareness and knowledge of CMV infection among women in Saudi Arabia, and to determine the characteristics of the study population that were associated with the overall CMV knowledge. This study may represent a baseline for awareness of the potential risks and preventative measures of CMV infection for pregnant women and may serve the basis for public awareness campaigns.

## Materials and methods

### Participants

The objective of the current study was to assess awareness and knowledge of CMV infection among women. We also aimed to evaluate the socio-demographic factors (age, educational level, occupation, place of residence, monthly household income, and family size) associated with knowledge of CMV infection. The study targeted women in all major regions of Saudi Arabia. The inclusion criteria required participants 1) to be 18 years of age or older; 2) to be Arabic speaking; 3) to sign a consent form to participate in the study. Participation in this study was voluntary with no compensation or incentives for participants.

### Questionnaire design and distribution

The present study used a structured, self-administered online questionnaire. The content of the questionnaire was designed based on validated questionnaires from previous studies evaluating the knowledge of CMV infection [28, 30, 31]. The questionnaire was initially developed in English and then translated to Arabic by the principal investigator and then back translated to English by experts in Microbiology with extensive clinical and academic experience of infectious diseases. The questionnaire consisted of three sections and a total of 21 questions (S1 Appendix). Section 1 (questions 1 through 9) was designed to gather sociodemographic data including age, marital status, place of residence, household income, family size, level of education, occupation, and pregnancy status. Section 2 (question 10 through 15) was intended to evaluate the general knowledge of CMV in which participants were asked a question regarding awareness of common childhood conditions including CMV infection. Women were then asked about modes of CMV transmission and symptoms. Knowledge of CMV modes of transmission was assessed by asking women to choose from nine choices of which six were correct. Knowledge about symptoms and clinical presentations of CMV was assessed by asking participants to choose from a total of ten answers of which eight clinical presentations of CMV were correct and two choices were incorrect. Section 3 (questions 16 through 20) contained questions related to CMV preventative measures and behaviors related to transmission of CMV.

Face and content validity had been determined to ensure whether the content of the questionnaire is relevant to the purpose of the study. The author clearly drafted the questionnaires’ items by conducting a thorough literature review and seeking expert opinion. The items were adapted from various published valid questionnaires [25, 28, 29, 31]. To establish the validity of the questionnaire, four experts in the areas of microbiology, virology, otolaryngology, and immunology were asked to review the drafted 21 items pertaining to CMV awareness, transmission, symptoms, and prevention. These experts were asked to rate the relevance of each item on the questionnaire to the conceptual framework using a 4-point Likert scale (1 = not relevant, 2 = somewhat relevant, 3 = relevant, 4 = very relevant). The responses of the experts were rated relevant and very relevant for all questionnaire items suggesting that the items are consistent with the conceptual framework.

The questionnaire used simple, clear, and unambiguous language in a way that ensures the participants provide responses that reflect the aim and objective of the study. To ensure that the content of the questionnaire was clear and understandable to participants, a pilot study using the questionnaire items was conducted on representative convenience sample of 20 women. These women were asked to answer the questions and rate the clarity and understandability of the questions on Likert scale from 0 (not clear) to 5 (very clear). Ambiguous questions that were missed or confused by many participants were examined and reworded. Based on feedback from the participants in the pilot study, one item was removed and a total of 20 questions were included in the final version of the questionnaire. To ensure reliability of the questionnaire, the same women were asked to complete it a second time. Analysis of the familiarity of the responses was measured using Cronbach’s alpha. The Cronbach’s alpha score was 0.74, indicating a good internal consistency of the items in the questionnaire.

### Data analysis

Statistical analyses were conducted using SPSS 24 for Windows (SPSS Inc, Chicago, IL). Categorical variables were reported in percentages and continuous variables were reported as mean and standard deviation. Binary logistic regression analyses were conducted to examine the association between CMV knowledge (dependent variable: heard of CMV previously) and demographic predictor variables (i.e., age, education, marital status, occupation, family size, monthly household income, pregnancy status). Odds ratios and 95% confidence intervals were reported. *P*-values less than or equal to 0.05 were considered statistically significant.

Among women who have heard of CMV, specific knowledge of CMV was categorized into three domains including modes of transmission, symptoms, and prevention. Within these three domains, correct responses were assigned a score of “1” and incorrect responses were assigned a score of “0”, and then summed for total scores of 6, 8, and 4 points, respectively. Knowledge of the transmission, symptoms and prevention domains were classified into “high” and “low” using a cut-off point of 70%.

### Ethical approval

This study was reviewed and approved by the institutional review board of the University of Hail (Protocol number: H-2020-001). Informed consent was obtained in the first page of the online questionnaire and participants were prompted to complete the questionnaire only after consenting to participate in the online questionnaire by clicking the “Continue” button. The prospective participants were not informed that CMV was the primary topic of the study as to not influence their answer to the question “have you ever heard of CMV previously”?, but that they were being surveyed about general health and knowledge of infections.

## Results

### Sociodemographic characteristics of participants

A total of 1008 women opened the online questionnaire and of these, 4 (0.39%) did not consent to participate and accordingly the final dataset was 1004 women. The sociodemographic characteristics of the study population are presented in Table 1 (also see S1 File for all dataset).

**Table 1:** Sociodemographic characteristics of survey respondents and their association with knowledge of CMV.

Sociodemographic characteristics	N (%)	OR	95% CI	*p*-value
**Age (Years)**				
18–30 (R)	456 (45.4)	1		
31–40	419 (41.7)	0.722	0.34–1.55	0.402
41–50	110 (11)	0.39	0.05–1.76	0.22
>50	19 (1.9)	0	0	0.99
**Region**				
Eastern (R)	461 (45.91)	1		
Western	165 (16.43)	1.703	0.65–4.49	0.328
Northern	133 (13.24)	1.082	0.36–3.29	0.889
Southern	54 (5.37)	1.507	0.34–6.64	0.588
Central	191 (19.02)	1.017	0.41–2.06	0.972
**Place of residence**				
Rural (R)	274 (27.29)	1		
Urban	730 (72.70)	0.358	0.17–0.78	0.007*
**Education**				
High school (R)	282 (28.09)	1		
Undergraduate	664 (66.14)	20.362	3.82–75.63	0.0001*
Master’s degree	46 (4.58)	49.370	6.63–265.77	0.0001*
Doctoral Degree	12 (1.19)	36.032	2.76–378.33	0.006*
**Employment**				
Unemployed (R)	557 (55.47)			
Student	134 (13.35)	0.5	0.18–1.42	0.996
Healthcare student	40 (3.98)	67.62	20.90–218.7	0.0001*
Healthcare practitioner	54 (5.38)	29.39	13.11–65.84	0.0001*
Governmental sector	181 (18.02)	0.36	0.10–1.28	0.114
Private sector	38 (3.78)	1.24	0.26–5.84	0.789
**Family monthly income**				
5000–15000 SR (R)	819 (81.57)	1		
15100–20000 SR	106 (10.56)	1.26	0.48–3.35	0.639
>20000 SR	79 (7.86)	1.33	0.47–3.74	0.589
**Marital status**				
Single (R)	181 (18.02)	1		
Married	823 (81.97)	0.94	0.26–3.54	0.92
**Family size**				
1–3 (R)	272 (27.09)	1		
4–8	343 (64.04)	1.22	0.57–2.63	0.64
> 8	89 (8.86)	0.96	0.19–4.68	0.96
**Pregnancy**				
Not pregnant (R)	917 (91.33)	1		
Pregnant	87 (8.66)	2.248	0.83–6.08	0.110

*Significance at 0.01

CI: confidence interval, OR: odds ratio, R: reference group, SR: Saud Riyals (1 SR = $ 0.27).

The mean age of participants was 32 years (standard deviation [SD] = 8.36) and about 69% of women were in the 18–35 years age group, the highest proportions of participants were from the Eastern region (45.91%) and 72.70% of the total sample reported themselves as urban residents. Most respondents (81.97%) were married while 181 (18.03%) were single with no childbearing experience. A total of 87 respondents (8.66%) were pregnant at the time of survey. The majority of women (71.94%) held undergraduate degree and above from which 66.14% hold undergraduate degree, followed by a master’s degree for 4.58% of respondents, and a Doctor of Philosophy (PhD) degree for 1.19% of respondents. More than a quarter (27.19%, n = 273) of respondents were employed with 21.80% of respondents reported working in non-healthcare professions, followed by 5.38% were health practitioners. Students constitute 17.33% (n = 174) of the total sample, of which 40 (3.98%) are college students in healthcare specialties. The majority of respondents (81.57%) had a monthly household income of about 5000 to 15000 Saudi Arabian Riyals (SR) compared to 15100–20000 SAR in 10.56%, and >20000 SAR in 7.86%. Nearly two-third of participants (64.04%) live in houses with 4–8 persons per household with the distribution of household of participants varied from 1–3 in 27.09%, and > 8 in 8.86%.

### Knowledge and awareness of CMV infection

**Fig 1:**
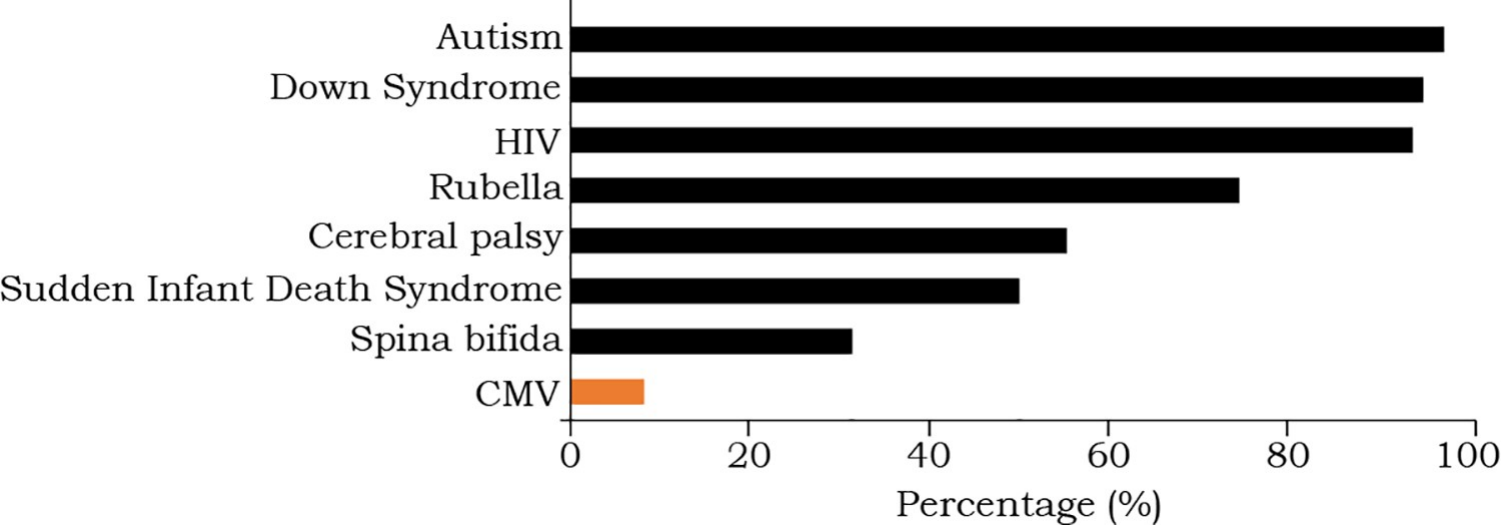
Survey respondents’ knowledge and awareness of various medical conditions.

When asked whether they have ever heard about common congenital conditions and birth defects including CMV, 82 of the 1004 surveyed women (8.17%) indicated that they had previously heard about CMV (Fig 1). Knowledge of CMV was ranked the lowest compared to knowledge of other birth defects and congenital diseases such as rubella (74.5%), HIV (93.82%), Down syndrome (95.01%), cerebral palsy (55.28%), sudden infant death syndrome (50%), autism (97.31%), and spina bifida (31.37%). Internet (37.25%) was the most common source of information about CMV, followed by attending university (36.27%), workplace (10.67), medical doctors (3.88%), social media (6.79%), and family member or a friend (4.85%).

The mean age of those who reported they had heard about CMV was 29.48 (SD = 7.4) years, and approximately two-third of participants held an undergraduate degree or higher (68.27%) and were in the 18–30 years age group (59.75%). In terms of employment, the highest proportion of participants were healthcare practitioners (n = 27, 32.92%) followed by college students (n = 25, 30.48%) of which 14 students were majoring in healthcare specialties (see S2 File for demographic characteristics and dataset of those who heard of CMV).

A binary logistic regression analysis was conducted to identify potential associations between knowledge of CMV and socio-demographic characteristics (Table 1). Knowledge of CMV was associated with educational attainment in which those with college degrees were more likely to have heard of CMV (Undergraduate: OR, 20.36; 95% CI, 3.82–75.63, *P* = 0.0001; Master’s degree: OR, 49.37; 95% CI, 6.63–265.77, *P* = 0.0001; Doctoral degree: OR, 36.03; 95% CI, 2.76–378.33, *P* = 0.006). With regards to employment, healthcare students (OR, 67.62; 95% CI, 20.90–218.7, *P* = 0.0001) and healthcare practitioners (OR, 29.39; 95% CI, 13.11–65.84, *P* = 0.0001) were more likely to have heard of CMV than those who worked in governmental and private sectors (Table 1). Urban residents had lower odds of knowledge about CMV (OR, 0.358; 95% CI, 0.17–0.78, *P* = 0.007) compared to rural residents. There was no significant difference in CMV awareness by pregnancy status, family size, marital status, monthly income, and region.

### Knowledge about transmission and symptoms of CMV infection

To assess women’s knowledge regarding mode of CMV transmission, women were presented with nine choices of which six were correct answers. Among women who reported having knowledge of CMV (n = 82), 19 participants (23.17%) reported that they “don’t know” how CMV is transmitted, 8 participants (9.75%) correctly identified all modes of transmission, 37 participants identified 1–3 correct answers (45.12%), and 18 participants identified 4–5 correct answers (21.95%) (Table 2). Concerning specific modes of CMV transmission, 59.75% were aware that CMV can be transmitted through mother-to-child before and immediately following birth, unprotected sex (48.78%), blood transfusion (36.58%), direct contact with body fluids of infected person (45.12%), touching eyes or inside the nose or mouth after coming in contact with body fluids of an infected person (31.70%), and breastfeeding (28.04%) (Table 2). In contrast, 2.43% and 10.97% of participants incorrectly identified air and eating undercooked food, respectively as possible modes of CMV transmission.

**Table 2:** Knowledge of CMV modes of transmission among survey respondents that previously heard of CMV.

Mode of transmission	N (%)
Touching your eyes or inside the nose or mouth after coming into contact with the body fluids of an infected person*	26 (31.70)
Eating undercooked meat	9 (10.97)
Placental transmission*	49 (59.75)
Direct contact with body fluids of infected person*	37 (45.12)
Sexual intercourse*	40 (48.78)
Blood transfusion*	30 (36.58)
Breastfeeding*	23 (28.04)
Air	2 (2.43)
Don’t know	19 (23.17)

* indicates correct answers

Knowledge about symptoms associated with congenital CMV was assessed by asking participants to choose from a total of 10 choices of which 8 possible common clinical presentations of CMV were correct and 2 choices were incorrect. Twenty-seven participants (32.92%) were completely unaware of the clinical symptoms of CMV infection (Table 3).

**Table 3:** Knowledge of symptoms of congenital CMV among survey respondents that previously heard of CMV.

Symptoms related to CMV	N (%)	No. of correct answers	N (%)
Hearing loss*	20 (24.39)	1–3 correct answers	33 (34.76)
Mental retardation*	28 (34.14)	4–7 correct answers	19 (23.17)
Microcephaly*	18 (21.95)	8 correct answers	3 (3.65)
Petechia*	17 (20.73)	Incorrect answers	15 (18.29)
Vision loss*	15 (18.29)		
Death*	31 (37.80)		
Heart defects	9 (10.97)		
Hepatomegaly*	26 (31.70)		
Splenomegaly*	21 (25.60)		
Cancer	6 (7.31)		
Don’t know	27 (32.92)		

* indicates correct answers

Although the remaining 55 participants reported knowledge of CMV-associated symptoms, their responses varied significantly. Only three women correctly identified all possible symptoms of CMV whereas 34.76% correctly identified 1–3 correct response, and 23.17% correctly identified 4–7 correct responses (Table 3). In terms of specific CMV-associated symptoms, women with knowledge of CMV correctly identified death (n = 31, 37.80%), mental retardation (n = 28, 34.14%), hearing loss (n = 20, 24.39%), petechiae (n = 17, 20.73%), microcephaly (n = 18, 21.95%), vision problems (n = 15, 18.29%), hepatomegaly (n = 26, 31.70%), and splenomegaly (n = 21, 25.60%) as CMV-associated symptoms, while 6 (7.31%) and 9 (10.97%) women incorrectly identified cancer and heart defects, respectively as symptoms of CMV (Table 3).

### Knowledge about preventative measures of CMV

About one-quarter of respondents (n = 22, 26.82%) reported they were not aware of preventative measures against CMV (Table 4).

**Table 4:** Knowledge of preventative measures against CMV among survey respondents that previously heard of CMV.

CMV preventative measures	N (%)
Hand washing*	35 (42.68)
Avoid sharing of utensils with children*	31 (37.80)
Avoid direct skin contact	13 (15.85)
Avoid contact with child’s bodily fluid*	37 (45.12)
Physical exercises	2 (2.43)
Avoid kissing children on the lips*	37 (45.12)
Avoid drinking soda and coffee	5 (6.09)
Vaccination	34 (41.46)
Don’t know	22 (26.82)

* indicates correct answers

Of the remaining 60 women who identified preventative measures, only 23 women (28.04%) correctly identified at least one of the possible preventative measures with no incorrect answers. In general, women were able to correctly identify the following preventative measures: hand washing (n = 35, 42.68%), avoid sharing of utensils and drinking cups with children (n =, 37.80%), avoiding contact with child’s bodily fluid (n = 37, 45.12%), and avoid kissing children on the lips (n = 37, 45.12%) (Table 4). By contrast, some women incorrectly identified the following as preventative measures against CMV: avoid direct skin contact (n = 13, 15.85%), avoid drinking soda and coffee during pregnancy (n = 5, 6.09%), and 34 women (41.46%) stated vaccination as protective measure against CMV (Table 4).

We also asked women if they had been provided with some instructions to protect themselves against CMV. Of the total surveyed women, 816 (81.27%) reported that they did not receive instructions form healthcare professionals on how to protect themselves from CMV infection while 178 (17.73%) did not know whether they were provided with protective instructions. Among those who reported they heard about CMV (n = 82), most of the respondents (n = 61, 74.39%) indicated that they were not provided with instructions to protect themselves against CMV. Overall, women who previously heard of CMV were able to identify more correct answers in the prevention domain of CMV than in the clinical symptoms and transmission domains. On average 15.80%, 11%, and 15.80% of women had high knowledge in the transmission, symptom, and prevention domains, respectively.

## Discussion

This survey study aimed to evaluate the awareness and knowledge of CMV infection among women in Saudi Arabia. CMV is considered as the leading intrauterine source of permanent disabilities among children such as mental retardation and hearing loss. Developed and developing countries reported high seropositive rates for CMV infection among women, e.g., US (58.3%; [32]), Europe (30–90%; [33]), India (80–90%; [34]), Pakistan (94.4%; [35]), Saudi Arabia (92–100%; [18]), and Africa (60–100%; [36]). Despite the high seropositive rate of CMV among women and the detrimental impact of CMV infection if contracted during the first trimester of pregnancy, there are several gaps in women’s knowledge of CMV [25, 26, 29, 30]. To the best of our knowledge, our study is the first that explores CMV in relation to awareness and knowledge of symptoms, modes of transmission, and risk and preventative behaviors among women in Saudi Arabia.

The current study revealed that Saudi women were less aware of CMV infection compared to other far less frequent intrauterine infectious diseases. In the participant’s answer to the question of the general knowledge of CMV infection, very few women (8.17%) stated they had previously heard about CMV. This level of knowledge is much lower than figures reported in previous studies from developed countries which ranges from 13–60% [25, 26, 28–30]). The dissimilarities between our findings and previous studies may exist due to the differences in the target population being investigated as most of previous studies recruited convenient samples of pregnant women or women visiting healthcare settings. Moreover, CMV knowledge in the current study was ranked the last compared to other less frequent infectious diseases causing congenital and birth defects, consistent with previous studies [25, 28, 29, 37, 38]. Our findings demonstrate the importance of educating women, particularly pregnant women, about CMV modes of transmission and primary preventive measures, a finding which has also been highlighted in previous studies [25].

This survey also revealed that the level of knowledge pertaining to CMV infection increased significantly among healthcare students and practitioners. Cordier et al. found a strong positive association between CMV awareness and knowledge of pregnant women employed in healthcare professions [30]. Nevertheless, the current study also revealed knowledge gaps with regards to certain modes of transmission and clinical symptoms even among respondents working in healthcare settings. Gynecologists less frequently include congenital CMV in their consultation visits with pregnant women [39]. In our study, only 1.10% of women were aware of being tested for CMV. Furthermore, most respondents (81.30%) reported that they were not provided with instructions at their regular doctor consultations during pregnancy. This finding is surprising since serological screening for TORCH infections for pregnant women is frequently performed during the antenatal period in the Saudi Ministry of Health public hospitals. These findings highlight some possible communication failures existing between healthcare practitioners and pregnant women. It may also indicate low knowledge of congenital CMV among healthcare practitioners in Saudi Arabia, a common finding reported in previous studies in France [30], Netherlands [40], and the United States [41]. For example, a survey study conducted in France showed that 46% of medical doctors did not know the transmission mode of CMV infection in newborns [30]. In the Netherlands, only half of medical doctors knew symptoms in newborns [40]. These findings have two important implications. First, efforts from healthcare practitioners should be directed toward providing sufficient instructions concerning lifestyle preventative behaviors to pregnant women or those who are planning for pregnancy. Second, additional efforts are required for healthcare practitioners to further update their knowledge related to CMV transmission, clinical symptoms, and lack of vaccine, CMV testing paradigms, and prevention of CMV. This can also be potentially achieved through a mandate disseminated by Saudi Ministry of Health to all public and private facilities to emphasize the topic of congenital CMV in continuing education programs for doctors caring for pregnant women and newborns.

The present study also demonstrated a relationship between women’s awareness and level of education and place of residence. Consistent with previous studies, the current study showed that education is an important predictor for knowledge and awareness of CMV infection in which women with bachelor’s degree or higher have more knowledge of CMV infection and its preventative measures. These findings have two important implications. First, community members with low educational attainment are the most appropriate target group in awareness programs to improve knowledge towards CMV in Saudi Arabia. Second, adequate information related to CMV used in awareness and prevention programs needs to be easily understood for individuals with low academic education. These findings indicate that public awareness and community-wide education programs are needed at all levels including those with low educational attainment, rural residence, and other socioeconomic factors.

With respect of age, the level of CMV knowledge and awareness of participants aged 18 to 40 years is higher, although not statistically significant, than those aged above 40 years. This trend is likely due to a relatively greater use of and access to Internet and social media platforms using smartphones in the young generation. Indeed, most women in the current study reported the Internet as one of the main sources of information related to CMV. These findings are in line with other studies that reported young women used the Internet to search for information related to CMV [29] and for other health-related information [42, 43]. This presents an opportunity for the Saudi authorities to use the internet as a powerful communication channel for health education programs to raise awareness of CMV infection. A potential approach is to develop a website (e,g., National CMV Foundation in the United States) that is linguistically and culturally appropriate for Saudi women aimed at raising awareness and promote education about the risks and prevention of congenital CMV. However, there are some caveats involved in using only online information for health education campaigns. The association between access to online information and socioeconomic status is consistent with the fact that the Internet tends to benefit those with higher socioeconomic status and those with higher educational attainment. Hence, awareness campaigns developed and supervised by the Saudi Ministry of Health through various, linguistically appropriate channels are needed.

A better awareness and knowledge of CMV’s modes of transmission is crucial for the prevention of CMV infection among pregnant women. This study demonstrated that women generally had poor knowledge of modes of CMV transmission. Among women who previously heard of CMV, 23.17% of respondents were not aware of CMV modes of transmission. In this context, only 49 women of the total sample were aware of CMV transmission via placental, maternal-to-fetus transmission (59.75%) and 23 women were aware of breastfeeding. Furthermore, other modes of CMV horizontal transmission were also less recognized, consistent with previous studies [25]. Specifically, very few women (n = 30) of the respondents were aware of contact with and exposure to body fluids of infected person through touching mouth and nose (45.12%) as a mode of CMV transmission. Altogether, these findings highlight that primary level prevention of both vertical and horizontal transmissions modes of CMV represent one of the main strategies to reduce the incidence and spread of CMV. This goal can be achieved if the public have adequate knowledge of the ways by which this virus is transmitted. Additionally, comprehensive participation and adherence is required from community members to practicing preventative measures concerned with vertical and horizontal transmission of the virus.

CMV infection in immunocompetent adult individuals is usually asymptomatic. However, approximately 10–15% of infected infants are born with CMV-related symptoms [9] while the remaining 90% of congenital CMV-infected infants are asymptomatic [12]. Our findings demonstrated that most women who stated they heard of CMV were not able to correctly identify all possible clinical presentations of CMV infection, with one-third (n = 27/82) of women reporting no knowledge of CMV-induced symptoms. Women were more familiar with mental retardation and death compared to other severe neurological consequences and multiorgan involvement in babies due to CMV infection. Furthermore, only one quarter of women (24.39%) reported hearing loss as CMV-associated clinical symptom. Congenital CMV is considered the most common infectious cause of sensorineural hearing loss in infants born with maternal primary CMV infection [14, 44]. Awareness campaign and health intervention programs should clearly communicate the severe complications to babies related to congenital CMV infection. Emphasis should be laid on the fact that CMV-infected children may appear well during early years of life, but may develop late-onset or progressive problems such as hearing loss.

Adequate knowledge of CMV preventative measures is essential for understanding and preventing horizontal and vertical transmission of CMV infection. About a quarter of women (n = 22) reported no knowledge of CMV preventative measures and about 45% gave incorrect answers pertaining to CMV preventative measures, and only 15.6% of surveyed women had good knowledge about the CMV preventative precautions. With regards to knowledge of specific preventative measures, our study revealed that 45% of women reported avoiding contact with body fluids of infected persons and avoid kissing children on the lips as preventative measures against CMV. Vaccination was incorrectly reported by 41% of women. Vaccination against CMV infection has not yet been developed to prevent CMV primary infection during pregnancy. This suggests that seronegative women should be clearly educated that the maternal-to-child transmission of CMV can only be reduced by practicing standard presentative measures until an effective vaccine against CMV is available [45, 46]. Furthermore, health education for congenital CMV infection should be considered early in the antenatal period. Maternal seroconversion, especially during the periconceptional periods or the first trimester of pregnancy, is associated with higher probability of transplacental transmission [47]. Furthermore, severe fetal CMV-related abnormalities are linked to maternal primary infection in the first trimester [48]. These findings highlight the fact that preventative measures against CMV maternal-to-child transmission should be ideally communicated to pregnant women early at their first prenatal visits and before conception for those planning pregnancy.

## Conclusion

Congenital CMV is recognized as a major public health concern in the world that results in severe medical complications and deaths than other maternal and intrauterine infections. The current study showed that the level of awareness and knowledge of CMV infection among Saudi women and more importantly women of childbearing age is significantly poor. In general, women demonstrated poor knowledge of CMV modes of transmission, CMV-associated clinical presentations, and preventative measures. These findings call for the implementation of well-designed awareness campaigns to increase awareness and knowledge of CMV as well as to promote CMV preventive practices among women of childbearing age.

## Supporting information

S1 Appendix Cytomegalovirus awareness and knowledge questionnaire in Arabic and English languages. (PDF)

S1 File Dataset and summary data for all study participants. (XLSX)

S2 File Dataset and summary data for women who had heard of cytomegalovirus. (XLSX)
