# Supplementary material for: Knowledge of cytomegalovirus infection among women in Saudi Arabia: A cross-sectional study
Source: PLoS One. 2022 Sep 29;17(9):e0274863. doi: 10.1371/journal.pone.0274863 (PMC9522269; doi:10.1371/journal.pone.0274863)
Supplement: S1 Appendix — (PDF) [file pone.0274863.s001.pdf]

Appendix: Questionnaire (Almishaal, 2021)

Knowledge and Awareness of Cytomegalovirus among Women

A- Socio-demographic characteristics

1- **What is your age? ______ Years**

2- **Marital status:**
- A- Single
- B- Married
- C- Divorced
- D- Widow

3- **Number of family members?**
- A- 1
- B- 2-3
- C- 4-6
- D- 7-8
- E- > 8

4- **What is the highest level of education you have completed?**
- A- No formal education
- B- Primary School
- C- Secondary school
- D- Diploma
- E- Bachelor’s degree
- F- Master’s degree
- G- Doctoral degree

5- **Occupation?**
- A- Unemployed
- B- Student
- C- Teacher
- D- Healthcare practitioner
- E- Engineer
- F- Private sector
- G- Other (please specify) ___________

6- **Which region do you live in?**
- A- Eastern region
- B- Western region
- C- Middle region
- D- Northern region
- E- Southern region

7- **Which area you live in?**
- A- Rural
- B- Urban

أ- الخصائص الاجتماعية والجغرافية

1- العمر: ____ سنة

2- الحالة الإحتماعية:
- أ- عزباء
- ب- متزوجة
- ت- مطلقة
- ث- أرملة

3- عدد أشخاص العائلة؟
- أ- ١
- أ- ٢-٣
- ب- ٤-٦
- ج- ٧-٨
- د- > ٨

4- ما هي أعلى شهادة حصلتي عليها؟
- أ- لم أتلقى أي تعليم رسمي
- ب- شهادة ابتدائية أو متوسطه
- ت- شهادة ثانوية
- ج- دبلوم
- ح- بكالوريوس
- د- ماجستير
- ذ- دكتوراة

5- الحالة الوظيفية:
- أ- غير موظفة
- ب- طالبة
- ت- معلمة
- ث- دبلوم
- ج- طبيبة أو من ضمن الكادر الصحي
- ح- مهندسة
- خ- قطاع خاص
- د- أخرى (أذكرها): ___________

6- ماهي المنطقة من مناطق المملكة التي تعيشين بها؟
- أ- المنطقة الشرقية
- ب- المنطقة الغربية
- ت- المنطقة الوسطى
- ث- المنطقة الشمالية
- ج- المنطقة الجنوبية

7- مكان المعيشة؟
- أ- مدينة
- ب- قرية

Appendix: Questionnaire (Almishaal, 2021)

Knowledge and Awareness of Cytomegalovirus among Women

8- Family monthly income (Saudi Riyals [SR])?

A- ≤5000 SR
B- 5000 – 10000 SR
C- 10100 – 15000 SR
D- 15100 – 20000 SR
E- ≥ 20000 SR

9- Are you pregnant?

A- Yes
B- No

8- الدخل الشهري للعائلة (بالريال السعودي)؟

أ- < ٥٠٠٠ ريال سعودي
ب- ٥٠٠٠ – ١٠٠٠٠ ريال سعودي
ت- ١٠١٠٠ – ١٥٠٠٠ ريال سعودي
ث- ١٥٠٠٠ – ٢ ٠٠٠ ريال سعودي
ج- ≤ ٢٠٠٠٠ ريال سعودي

9- هل أنتي حامل؟

أ- نعم
ب- لا

B- Health status characteristics

ب- خصائص الحالة الصحية

10- Have you ever heard about any of the following medical conditions (choose all that apply)?

A- Cerebral palsy
B- Cytomegalovirus (CMV)
C- Rubella
D- Down Syndrome
E- Sudden Infant Death Syndrome
F- Spina bifida
G- Autism
H- HIV

11- Have you been provided with any information regarding Cytomegalovirus?

A. Yes
B. No

12- If you answered yes to question 11, What is the source of this information (choose all that apply)?

A- OB-GYN
B- Pediatrician
C- General practitioner
D- Social media
E- Internet
F- A family member or a friend
G- Social media
H- Workplace
I- University

10- هل سبق سمعتي عن الأمراض التالية (يمكن الإختيار أكثر من إجابة) ؟

أ- الشلل الدماغي
ب-الفيروس المضخم للخلايا (باللغة الإنجليزية: سايتو ميغالو فيروس أو اختصاراً سي ام في – CMV)
ت-الحصبة الألمانية
ث-متلازمة داون
ج- متلازمة موت الرضع المفاجئ
ح- الشوك المشقوق أو الصلب المشقوق (وجود فتحة في الظهر خلقية)
خ- التوحد
د- الأيدز

11- هل تم تنبيهك أو تزويدك بأية معلومات عن الفيروس المضخم للخلايا (بالإنجليزية: سايتو ميغالو فيروس او اختصاراً سي ام في – CMV)؟

أ- نعم
ب- لا

12- إذا كانت الإجابة بنعم، ما هو مصدر المعلومات (يمكن الإختيار أكثر من إجابة)؟

أ- طبيب/ة النساء والولادة
ب-طبيب الأطفال
ت-طبيب عام
ث-الإنترنت
ج- مصادر التواصل الأجتماعي
ح- عن طريق أحد أفراد العائلة أوصديق
خ- مقر العمل
د- الجامعة

Appendix: Questionnaire (Almishaal, 2021)

Knowledge and Awareness of Cytomegalovirus among Women

13- In your opinion, what are the modes of transmission of CMV infection (choose all that apply)?

A- Through air
B- Direct contact with the skin of an infected person
C- Eating raw meat
D- Placental transmission to fetus
E- Exposure to body fluids
F- Sexual intercourse
G- Blood transfusion
H- Breastfeeding
I- I don't know

14- Do you have any information about the symptoms of Cytomegalovirus infection in newborn (congenital CMV ?

A- Yes
B- No

15- If yes, what are these symptoms of congenital CMV? (you can choose more than one answer)

A- Mental retardation
B- Hearing loss
C- Vision loss
D- Microcephaly
E- Hepatomegaly
F- Splenomegaly
G- Petechiae
H- Cancer
I- Heart defect
J- Death
K- I don't know

16- Have you been provided with any information regarding preventative measures against Cytomegalovirus?

 Yes
 No

13- هل لديك أي خلفية عن طرق انتقال الفيروس المضخم للخلايا (باللغة الانجليزية: سايتوميغالو فيروس أو اختصاراً سي ام في)، اختر من الاتي (يمكن الإختيار أكثر من إجابة)؟

أ- عن طريق الهواء
ب- عن طريق لمس عينيك أو داخل الأنف أو الفم بعد ملامسة سوائل الجسم لشخص مصاب
ت - عن طريق ملامسة جسد المصاب بالفيروس المضخم للخلايا أو مصافحته
ث - عن طريق أكل اللحوم الغير ناضجة
ج - عن طريق المشيمة من الأم لجنينها
ح - عن طريق سوائل الجسم مثل حليب الأم، اللعاب المخاط الأنفي أو دموع الشخص المصاب
خ - عن طريق ممارسة العملية الجنسية مع شخص مصاب بالفيروس
د -عن طريق نقل الدم بحيث يكون الشخص المتبرع مصابا بالفيروس
ذ- عن طريق حليب الثدي من أم مصابة لطفلها
ر- لا أعرف

14- هل لديك أي علم عن أعراض الفيروس المضخم للخلايا (سايتوميغالوفيروس-سي ام في الخلقي) عند الأطفال حديثي الولادة (العدوى الخلقية)؟

أ- نعم
ب-لا

15- إذا كانت الإجابة بنعم، ما هي بعض هذه الأعراض، أختر من الاتي (يمكن الإختيار أكثر من إجابة)؟

أ- تخلف عقلي
ب- ضعف سمع
ت- مشاكل في النظر
ث- محيط رأس صغير
ج- تضخم الكبد
ح- تضخم الطحال
خ- بقعًا بنفسجية أو طفحًا جلديًا
د- أورام سرطانية
ذ- موت
ر- لا أعرف

16- هل لديك خلفية أو تم تزويدك بمعلومات إحترازية للوقاية من عدوى الفيروس المضخم للخلايا (سايتو ميغالوفيروس-سي ام في) ؟

أ- نعم
ب- لا

Appendix: Questionnaire (Almishaal, 2021)

Knowledge and Awareness of Cytomegalovirus among Women

17- If you answered yes, what is the source of these preventative measures (choose all that apply)?

A- OB-GYN
B- Pediatrician
C- General practitioner
D- Nurse
E- Social media
F- Internet
G- A family member or a friend
H- Workplace
I- University

18- In your opinion, what are these preventative measures against CMV infection?

A- I have no knowledge of preventative precautions
B- Washing hands thoroughly after changing your child's diaper
C- Avoid sharing utensils (spoons, cups) with your children
D- Avoid touching the skin (superficial) of your children
E- Avoid contact with your child's body fluids
F- Exercising during pregnancy
G- Avoid kissing your child on the lips
H- Avoid drinking coffee and soda during pregnancy
I- Getting the required immunization during pregnancy
J- I don't know

19- Have you ever been tested for CMV?

A- Yes
B- No
C- I don't know

20- Have you received further information about CMV following the test?

A- Yes
B- No
C- I don't know

17- إذا كانت الإجابة بنعم، ما هو مصدر هذه الإجراءات الإحترازية؟

أ- طبيب/ة النساء والولادة
ب- طبيب الأطفال
ت- طبيب عام
ث- ممرضة
ج- الإنترنت
ح- مصادر التواصل الأجتماعي
خ- عن طريق أحد أفراد العائلة أوصديق
د- مقر العمل
ذ- الجامعة

18- في رأيك، ما هي هذه الطرق الإحترازية لتقليل فرصة إصابتك بالفيروس المضخم للخلايا (يمكن الإختيار أكثر من إجابة)؟

أ- غسل اليدين جيدا بعد الإنتهاء من إطعام الأطفال أوتغيير حفاضات الطفل
ب- تجنب إستخدام أدوات الأكل (الملاعق والأكواب) التي يستخدمها الطفل، وكذلك مناشف السباحة
ت- تجنب ملامسة جسد الطفل (الملامسة الخارجية لجلده) المصاب بالفيروس
ث- تجنب ملامسة سوائل الطفل كلعابه، دموعه، المخاط الأنفي
ج- أداء التمارين الرياضية أثناء الحمل
ح- الإبتعاد عن تقبيل الطفل في الفم
خ- الإبتعاد عن شرب المشروبات الغازية أو القهوة بكثرة أثناء فترة الحمل
د- عن طريق أخذ التطعيمات اللازمة لهذا المرض أثناء فترة الحمل
ذ- لا أعلم

19- هل تم فحصك للفيروس المضخم للخلايا (سايتوميغالوفيروس-سي ام في)

أ- نعم
ب- لا
ت- لا أعلم

20- هل تم تزويدك بمعلومات تخص الفيروس المضخم للخلايا (سايتوميغالوفيروس) بعد الإختبار؟

أ- نعم
ب- لا
ت- لا أعلم
